# Body Temperature Patterns and Rhythmicity in Free-Ranging Subterranean Damaraland Mole-Rats, *Fukomys damarensis*

**DOI:** 10.1371/journal.pone.0026346

**Published:** 2011-10-18

**Authors:** Sonja Streicher, Justin G. Boyles, Maria K. Oosthuizen, Nigel C. Bennett

**Affiliations:** 1 Mammal Research Institute, Department of Zoology and Entomology, University of Pretoria, Pretoria, South Africa; 2 Department of Zoology, King Saud University, Riyadh, Saudi Arabia; Hokkaido University, Japan

## Abstract

Body temperature (T_b_) is an important physiological component that affects endotherms from the cellular to whole organism level, but measurements of T_b_ in the field have been noticeably skewed towards heterothermic species and seasonal comparisons are largely lacking. Thus, we investigated patterns of T_b_ patterns in a homeothermic, free-ranging small mammal, the Damaraland mole-rat (*Fukomys damarensis*) during both the summer and winter. Variation in T_b_ was significantly greater during winter than summer, and greater among males than females. Interestingly, body mass had only a small effect on variation in T_b_ and there was no consistent pattern relating ambient temperature to variation in T_b_. Generally speaking, it appears that variation in T_b_ patterns varies between seasons in much the same way as in heterothermic species, just to a lesser degree. Both cosinor analysis and Fast Fourier Transform analysis revealed substantial individual variation in T_b_ rhythms, even within a single colony. Some individuals had no T_b_ rhythms, while others appeared to exhibit multiple rhythms. These data corroborate previous laboratory work showing multiplicity of rhythms in mole-rats and suggest the variation seen in the laboratory is a true indicator of the variation seen in the wild.

## Introduction

Body temperature (T_b_) is an important physiological parameter that strongly affects fitness [1], [2]. Maintaining a high and constant T_b_ has long been thought to be an advantage for endotherms because, for example, a number of significant enzymes are heat-activated and chemical reaction rates are strongly tied to temperature [2]. However, it is energetically costly to maintain a high and constant T_b_ and thus variation in T_b_ is likely a universal phenomenon among endotherms [1], [3]. In fact, it is increasingly being argued this variation in T_b_ is an adaptive response to past selective pressures and the present local environment (see [1] for a review of the topic). While a large number of empirical studies have been done on homeothermic species in the wild [4], [5], [6], and some of these can be interpreted in such a way as to support the hypothesis of adaptive thermoregulation [7], [8], [9], [10], relatively few studies have explicitly addressed the possibility of adaptive responses in thermoregulation in relatively homeothermic species [1]. Notable classic examples that do included the work of Schmidt-Nielsen on camels [11] and recent examples of such studies are the those of Hetem et al. [12] on springbok (*Antidorcas marsupialis*) with three colour morphs and Glanville and Seebacher [13] on bush rats (*Rattus fuscipes*) during summer and winter. Hetem et al. [12] showed that black springbok, which are most likely to experience heat stress, had larger variations in T_b_ during the summer days, whereas white springbok, which are most likely to experience cold stress, had larger variations in T_b_ during winter days. Similarly, T_b_ variation was larger during winter than summer in bush rats in the wild [13].

Within the mammalian order Rodentia, there is great variation in T_b_ patterns, but subterranean species often regulate T_b_ at approximately 34°C [14], [15], [16]. Mole-rats are a family of subterranean rodents that inhabit sub-Saharan Africa and, with the exception of the naked mole-rat [17], are considered relatively homeothermic (while some species show drops in T_b_ under thermal stress in the laboratory, it is unclear if these drops are controlled). Although T_b_ has been measured for most mole-rat species, the focus of previous studies has typically not been to quantify T_b_ patterns *per se*, but rather to measure metabolic rates across a range of ambient temperatures (T_a_)(e.g., [18], [19], [20], [21]). Thus, available T_b_ data for mole-rats generally consist of either instantaneous measures of T_b_ during metabolic measurements or short term measures of T_b_ under artificial laboratory conditions (e.g., [16], [17], [22]). The T_b_ patterns of free-ranging animals have not been described for any mole-rat species in the field, even though they have been suggested as an ideal group for the study of thermoregulation and the evolution of endothermy [23].

We measured T_b_ in free-ranging Damaraland mole-rats (*Fukomys damarensis*) during summer and winter with two goals: a) these data provide the first measures of T_b_ in free-ranging mole-rats, a group whose thermoregulatory characteristics have been well-studied under laboratory conditions. While laboratory studies allow for precise control of environmental variables, recent evidence suggests that thermoregulatory characteristics often vary between wild and captive animals [24]. And b) this study allows for an additional empirical evaluation of the prediction that increased costs of thermoregulation associated with low T_a_s in conjunction with low energy availability (e.g., during winter) should lead to increased variation in T_b_ in homeotherms, just as it does in heterotherms [1], [25].

## Materials and Methods

### Ethics statement

All animal procedures were conducted by licensed veterinarians and approved by the Animal Use and Care Committee at the University of Pretoria (AO27/06).

### Study species and study area

The Damaraland mole-rat is a eusocial species that occurs in colonies of up to 40 individuals [26], [27]. They inhabit closed burrow systems which have a muted and tight temperature rhythm that is markedly different from surface temperature profiles [26], [28]. Previous studies have shown that Damaraland mole-rats have a core T_b_ of approximately 35°C [22], [29], [30], [31], which is characteristic for subterranean rodents [14]. Their thermoneutral zone ranges between 27–31°C and they are generally described as having good thermoregulatory capacities [22].

We conducted the study near Hotazel (27°17′S; 22°58′E) in the Northern Cape Province, South Africa during austral summer (December-March) of 2006–2007 and austral winter (April-September) of 2007. The study site is in the arid Kalahari region, which is characterized by semi-desert conditions including low annual precipitation and large daily fluctuations in T_a_. Average minimum daily temperatures from 1998–2010 at the Kathu, South Africa weather station were 3.9°C and 20.3°C for July and December, respectively. Average maximum daily temperatures were 19.0°C and 32.5°C for July and December, respectively. We captured animals with modified Hickman live traps using sweet potato as bait (Hickman, 1979). Traps were placed at an opening to the burrow system and covered with a layer of soil to prevent any light from entering the tunnel and to keep the traps cool. Traps were checked every two to four hours. When possible, all the members of a particular colony were trapped out before they were processed and released. A colony was considered completely captured when all animals including the reproductive pair were captured and no further animals came to the live traps. In order to verify that no animals remained in the burrow, the burrow system was dug back approximately 1 m to ensure there was not internal blocking. Before release, we housed animals in plastic containers 30 cm×60 cm×30 cm, where the floor had been covered with a layer of soil. Animals were fed an *ad libitum* diet of sweet potato which provides all necessary nutrients and water.

### Experimental procedures and temperature measurements

Once a colony had been captured, we surgically implanted a calibrated temperature datalogger (DS1922L iButtons, Maxim Integrated Products, Dallas, TX, USA) into the abdomen of each animal. The smallest individual implanted weighed 81 g, so the datalogger (3.2 g) was far less than 5% of body mass in all individuals. The iButtons were programmed to record temperature hourly with a resolution of 0.05°C. Dataloggers were covered in wax and sterilized in hibothane alcohol prior to insertion into animals. For the procedure, we anaesthetized each animal with ketamine hydrochloride (4–6 mg/kg) and medetomodine (0.06–0.15 mg/kg). After each procedure, we administered buprenorphine (0.05–0.1 mg/kg) for post-surgery analgesia, synulox (0.2 mg/kg) to avoid surgery related infections and atipamezole (0.3–0.7 mg/kg) to reverse the effects of medetomodine. Aseptic techniques were applied throughout the procedures. Animals were given 24 hours to recover from surgery before they were released back into their respective burrows. After four months (summer) and six months (winter), animals were recaptured and the dataloggers were removed surgically using the same procedures as described above. Different animals were used for each period since we had difficulty recapturing the same animals both seasons. Representative raw data of T_b_ are including in Table S1.

Mole-rats spend up to 80% of their time in the nest resting [32], so the environmental temperature experienced by mole-rats is essentially soil temperature, as opposed to actual air temperature [26]. Therefore, we measured soil temperatures using iButtons placed at 3 different depths: 0.5 m, 1.0 m, and 2.0 m in two pits approximately 1 km apart. These soil depths cover the range of depths at which mole-rats construct burrow structures (∼1.5 m) and nests (∼2.0 m) [26], [28]. Soil temperatures were recorded hourly during the summer and bihourly during the winter. All studies were conducted under permit number 0092/07 from the Northern Cape Department of Nature and Environmental Conservation.

### Data analysis

We calculated the mean, minimum, maximum, and variation in T_b_s for each 24 hour period. To quantify the variation in T_b_, we used the Heterothermy Index (HI) of Boyles *et al*. [33]:
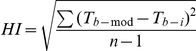
where T_b-mod_ is the modal T_b_, T_b-i_ is the T_b_ measurement at time *i* and *n* is the number of times T_b_ is sampled. The HI quantifies deviation away from the theoretically optimal temperature for performance as approximated by T_b-mod_. T_b-mod_ was calculated as the modal T_b_ for individuals that displayed unimodal distributions of T_b_ and the mode of the highest peak for individuals that displayed bimodal distributions of T_b_ [34], [35]. HI values were calculated for each animal over each 24 hr period [33]. We used repeated-measures ANOVAs to test the effects of season, gender, and body mass on the HI values and T_b_ characteristics measured. We used days as the repeated measure within each individual, which was nested within groups to account for non-independence caused by multiple individuals being sampled from a group. We also attempted to include average soil temperature at 2 m (approximately the depth of the burrows) on HI values, but the effect was insignificant in all derivations of the model, so we left it out of the final model for simplicity. We ran separate models with each T_b_ characteristic as the response variable using the PROC MIXED function in SAS (Version 9.2, SAS Inc., Cary, NC, USA) with a type-I error rate of 0.05. To model correlation within experimental units across time and between experimental units, we first determined the appropriate covariance structure for each dataset based on Akaike Information Criterion adjusted for small sizes (AICc) values [36]. We investigated differences between main effects using Fisher's Least Significant Difference Tests (LSD) assuming a type-I error rate of 0.05. When interactions occurred, we performed tests of main effects using the SLICE option in the LSMEANS statement [37], [38]. We used the Kenward-Roger method to estimate the degrees of freedom [39]. In addition, we fit linear and quadratic curves to the raw daily HI values to determine if HI values changed predictably across the season. All data are presented as mean ± SD.

We used cosinor analysis [40] to determine if any 24 hr rhythms of T_b_ were present in free-ranging mole-rats. We assumed 24 hr rhythms because these animals are exposed to 24 hr variations in burrow temperature [28]. For each animal, we also calculated percentage rhythm, i.e., the percentage of the variability in the data that could be accounted for by the fitted curve. As a complement to cosinor analyses, we used spectral analyses to detect possible rhythmic patterns outside the predicted 24 hour pattern. We used a smoothed periodogram based on a Fast Fourier Transformation (FFT) to describe the spectral density over the full range of frequencies [41]. We constructed one periodogram for each animal. Statistical analyses on T_b_ rhythms were carried out using R version 2.11.0 (http://www.r-project.org) and the cosinor analyses using the program Chrono2 (J.W.H. Ferguson, University of Pretoria).

## Results

Across the entire summer, the average soil temperatures decreased with increasing depth: 31.1°C±0.5 at 0.5 m; 29.7°C±0.3 at 1.0 m; and 27.5°C±0.4 at 2 m. This pattern was reversed during the winter as average temperatures increased with depth: 17.5°C±2.5 at 0.5 m; 19.4°C±2.4 at 1.0 m; and 21.3°C±2.2 at 2 m. The daily variation in soil temperature was small and similar between summer and winter. The mean daily standard deviation was 0.15, 0.03, and 0.01°C during summer at 0.5 m, 1 m, and 2 m, respectively and 0.16, 0.04, and 0.02 during winter, respectively. During the summer sampling period, the soil temperature increased throughout the season, while during winter, it decreased throughout the season.

**Figure 1:**
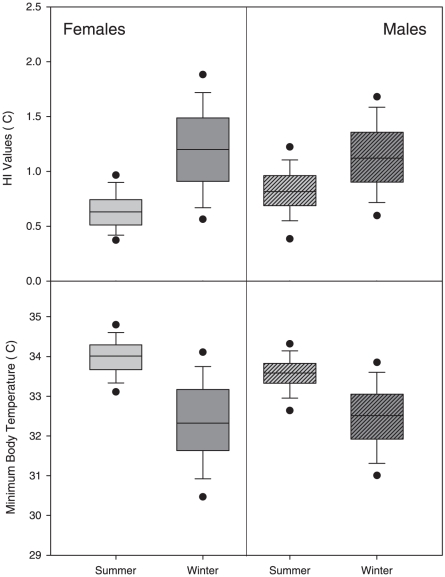
Heterothermy Indices (HI) and minimum body temperature for Damaraland mole-rats (*Fukomys damarensis*) during summer and winter in the Kalahari desert, South Africa.

During summer, 26 animals (10 males; 16 females) were captured and implanted with dataloggers and eight (2 males; 6 females) were recaptured. In the winter sampling period, 44 individuals were implanted (24 males; 20 females) and 15 were recaptured (9 males; 6 females). As indicated by HI values, Damaraland mole-rats allowed T_b_s to vary significantly more during winter (1.16°C±0.01) than summer (0.69°C±0.01; P = 0.002) and males (1.10°C±0.01) allowed T_b_s to vary significantly more than females (1.01°C±0.01; P = 0.027)(Fig. 1). The gender × season interaction was also significant (P = 0.003) and was driven by a larger change in HI values from winter to summer among females (1.21°C±0.01 vs. 0.65°C±0.01) than among males (1.13°C±0.01 vs. 0.82°C±0.02). HI values were not significantly related to body mass (P = 0.35), but the mass × season interaction was significant (P = 0.012) and was driven by a more strongly negative relationship between body mass and HI values during winter than during summer. Mean T_b_s were higher for both genders during summer (P<0.0001) and dropped more among females between summer and winter (35.05°C±0.01 vs. 34.67°C±0.01) than among males (34.74°C±0.02 vs. 34.62°C±0.008). Maximum T_b_s varied seasonally in the same pattern as mean T_b_s (data not shown), but the pattern in mean T_b_ was most strongly driven by minimum T_b_s. Minimum T_b_s were significantly higher during summer (33.86°C±0.02) than winter (32.42°C±0.02; P<0.0001) and among females (32.91°C±0.03) than males (32.59°C±0.02; P = 0.0003). The gender × season interaction was also significant (P<0.0001) and driven by a larger drop in minimum T_b_ from summer to winter among females (33.97°C±0.02 vs. 32.33°C±0.04) than males (33.56°C±0.03 vs. 32.47°C±0.02). During winter, the recorded minimum T_b_ dropped below 31°C in all but one individual and below 30°C in all but four individuals.

Among both genders during winter, the largest HI values occurred during mid-winter and the quadratic term was significant (P<0.0001). During the summer, the quadratic term was significant only among females (P<0.0001), with the smallest HI values occurring during mid-summer. However, in all four gender/season groups, AICc values indicate that linear curves fit the data better than do quadratic curves (ΔAICc<2 in all cases), so the small increases in fit associated with the quadratic model do not warrant the increase in complexity. The slope of the linear model, while significant in all four groups because of the large sample sizes (all P<0.009), was very near zero in all cases (all slopes were between −0.006 and 0.002). HI values increased slightly throughout winter among both genders. During summer, HI values increased across the season among males, but decreased among females. This difference in responses among males and females during summer explain the non-significant relationships between soil temperature and HI values in our initial model.

**Figure 2:**
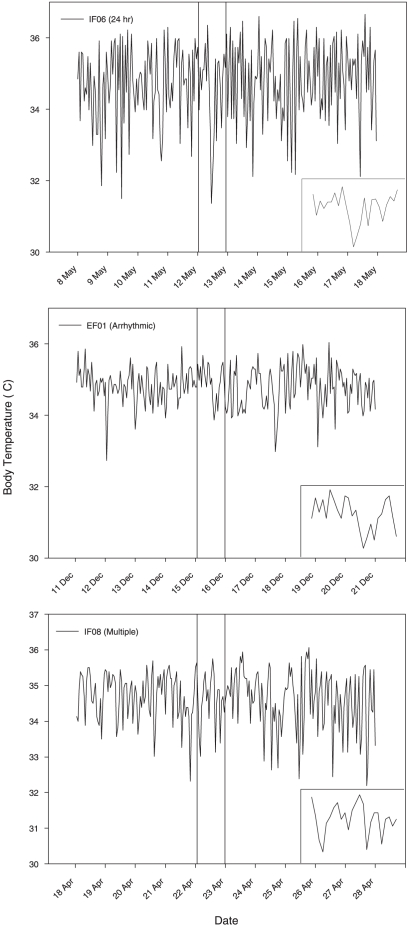
Examples of 10 day body temperature tracings for three distinct profiles in Damaraland mole-rats (*Fukomys damarensis*): (a) an animal with a 24 hour body temperature rhythm, (b) no body temperature rhythm, and (c) multiple body temperature rhythms. The vertical lines demarcate the 24 hr period displayed in the inset.

Both the cosinor and FFT analyses suggest considerable variation exists in rhythmicity of T_b_ cycles, with no overall pattern prevailing (Table 1). Some individuals exhibited 24 hour patterns of T_b_, while many other individuals displayed two rhythms (24 and 12 hour rhythms)(Fig. 2). Seven individuals were arrhythmic while other individuals displayed multiple rhythms. Interestingly, individuals within the same colony often had different T_b_ patterns.

**Table 1:** Descriptive statistics of rhythmicity in free-ranging Damaraland mole-rats (*Fukomys damarensis*).

Individual	Season	Spectral Rhythm	Mean Daily T_b_ (°C)	Mean Max Daily T_b_ (°C)	Mean Min Daily T_b_ (°C)	Mesor (°C)	Amplitude (°C)	Percentage rhythm	Zero-amplitude test	*P*-value
DF01	Summer	24 H	35.41	36.25	34.53	35.41	0.08	1.58%	*F*_2,2157_ = 17.27	*P<0.001*
DM02	Summer	24 H & 12 H	34.68	35.80	33.50	34.68	0.006	0.01%	*F*_2,2157_ = 0.06	*P = 0.94*
DM03	Summer	24 H & 12 H	34.8	36.08	33.61	34.8	0.05	0.22%	*F*_2,2157_ = 2.32	*P = 0.1*
EF01	Summer	arrhythmic	34.91	36.05	33.75	34.91	0.04	0.15%	*F*_2,2157_ = 1.64	*P = 0.19*
EF03	Summer	arrhythmic	34.91	36.11	33.88	34.91	0.05	0.26%	*F*_2,2157_ = 2.84	*P = 0.06*
EF04	Summer	24 H	35.03	36.25	33.82	35.03	0.02	0.24%	*F*_2,2157_ = 2.58	*P = 0.08*
EF07	Summer	arrhythmic	35.01	36.15	33.87	35.01	0.04	0.66%	*F*_2,2157_ = 7.11	*P<0.001*
EF08	Summer	24 H & 12 H	35.01	36.49	33.94	35.01	0.08	0.02%	*F*_2,2157_ = 0.24	*P = 0.79*
IF04	Winter	multiple	34.5	36.17	31.70	34.5	0.1	0.34%	*F_2,3957_* = 6.71	*P = 0.001*
IF06	Winter	24 H	34.72	36.21	32.42	34.72	0.13	0.75%	*F_2,3957_* = 14.96	*P<0.001*
IF08	Winter	multiple	34.67	36.15	32.36	34.67	0.08	0.26%	*F_2,3957_* = 5.17	*P = 0.006*
IM05	Winter	multiple	34.61	36.20	32.14	34.61	0.14	0.79%	*F_2,3957_* = 15.76	*P<0.001*
IM10	Winter	arrhythmic	34.65	36.18	32.21	34.65	0.1	0.46%	*F_2,3957_* = 9.13	*P<0.001*
IM11	Winter	multiple	34.71	36.18	32.65	34.71	0.09	0.43%	*F_2,3957_* = 8.58	*P<0.001*
IM13	Winter	arrhythmic	34.81	36.22	32.75	34.81	0.05	0.13%	*F_2,3957_* = 2.64	*P = 0.07*
IM14	Winter	multiple	34.72	35.99	32.91	34.72	0.08	0.48%	*F_2,3957_* = 9.57	*P<0.001*
JF01	Winter	multiple	34.52	36.19	32.01	34.52	0.03	0.04%	*F_2,3957_* = 0.77	*P = 0.46*
JF04	Winter	6 H	35.08	36.15	33.38	35.08	0.005	0.00%	*F_2,3957_* = 0.03	*P = 0.97*
JF06	Winter	24 H	34.5	36.05	32.10	34.5	0.09	0.33%	*F_2,3957_* = 6.6	*P<0.001*
JM02	Winter	arrhythmic	34.65	36.20	32.32	34.65	0.02	0.02%	*F_2,3957_* = 0.34	*P = 0.71*
JM03	Winter	multiple	34.51	35.90	32.65	34.51	0.04	0.09%	*F_2,3957_* = 1.82	*P = 0.16*
EMX	Winter	24 H & 12 H	34.36	36.04	31.91	34.36	0.09	0.28%	*F_2,3957_* = 5.61	*P = 0.004*
GM06	Winter	arrhythmic	34.59	35.91	32.72	34.59	0.05	0.15%	*F_2,3957_* = 2.94	*P = 0.05*

## Discussion

The patterns in T_b_ we recorded in free-ranging Damaraland mole-rats supported the predictions that variation in T_b_ should increase as the cost of thermoregulation increased and the benefit of maintaining strictly constant T_b_s decreased. Both genders allowed T_b_ to vary more during winter than during summer when soil temperatures at burrow level were lower. There were small changes in HI values across seasons, but interestingly, soil temperature was not a good predictor of HI values. Although the seasonal changes in HI values and T_b_ are not as large as in heterothermic species (e.g., [42]), they follow the same general pattern and we suggest the relatively small differences may be biologically important when considered in the context of energy expenditure over the course of an entire season. The seasonal patterns in HI values in Damaraland mole-rats were most strongly driven by changes in minimum T_b_, which decreased to as low as 28.5°C in some individuals. While individuals displaying these T_b_s would likely be considered torpid using many common metrics [34], [43], [44], there is no evidence to date that any mole-rat species uses torpor or hibernation, although in the laboratory, Damaraland mole-rats can occasionally be cold to the touch and can take several minutes to awake if disturbed (N.C. Bennett, pers. obs.). Importantly, the T_b_ fluctuations in Damaraland mole-rats are not exactly the same as those displayed by facultative heterotherms, which tend to maintain a constant, lowered set point during torpor. Variation in maximum T_b_ was much more constrained with T_b_ rarely exceeding 37°C. This corroborates previous suggestions that subterranean rodents may be at high risk of overheating and therefore carefully regulate any increases in T_b_ [14]. The HI values and T_b_ characteristics recorded herein were quite similar to those recorded in two other mole-rat species in the laboratory [45].

While estimates of energy expenditure are difficult based on T_b_ datasets, some conclusions can still be drawn. The soil temperatures recorded during summer were in the thermoneutral zone (TNZ) for Damaraland mole-rats while the soil temperatures during winter were considerably below TNZ for much of the winter [22]. In practice, this means that metabolic rates during late winter would be 2–3 times higher than during summer if Damaraland mole-rats attempt to maintain a relatively constant T_b_ throughout the year [22]. However, the low T_b_ values we recorded are similar to other subterranean mammals [46] and suggest that Damaraland mole-rats are using some form of apparently controlled bouts of hypothermia during winter. Even small decreases in the T_b_-T_a_ differential may greatly reduce energy expenditure and may be vital to survival.

In the endotherm literature, T_b_ is generally considered in the context of energy expenditure; however, there is also evidence that T_b_ affects performance in endotherms, as has been repeatedly shown in ectotherms [47]. Furthermore, it has been predicted that thermoregulatory patterns and the sensitivity of thermal performance should be co-adapted in endotherms [1]. In other words, heterothermic species should be able to maintain some performance across a wide range of T_b_s, while strict homeotherms should experience substantial decreases in performance in response to even relatively small changes in T_b_. In humans (i.e., strict homeotherms), every 1°C decrease in muscle temperature leads to a 2–5% decrease in performance [47] while highly heterothermic round-tailed ground squirrels (*Spermophilus tereticaudus*) showed no change in whole organism performance across an approximately 12°C range of T_b_s [48]. Given the relatively homeothermic patterns usually displayed by Damaraland mole-rats, some of the T_b_ fluctuations we recorded during winter in this study may be large enough to lead to substantial decreases in performance. Conversely, T_b_ is known to be correlated with activity in mole-rats [15], so these decreases in T_b_ may impose a relatively low performance cost if activity is already down regulated. An interesting avenue of future research will be to evaluate the effects of these fluctuations on everything from predator avoidance (e.g., running speed; [48]) to reproductive efficiency in highly homeothermic mammals such as mole-rats.

Damaraland mole-rats do not exhibit clear T_b_ rhythms as is the case for many rodents [15], [49], [50], [51]. Instead, a variety of T_b_ rhythms were found among Damaraland mole-rats, ranging from arrhythmic to 24 hour rhythms. A 24 hour T_b_ rhythm can easily be explained [52] and a 12 hour T_b_ rhythm may correspond to the amount of light in a day. Given that both these rhythms are caused by the Earth's rotation, it seems plausible that a single animal could display both of these rhythms. The multiple rhythms that some mole-rats displayed are much more challenging to explain. Various biological rhythms exist within the body, from activity rhythms to hormone rhythms [51], [53], [54] and any number of these biological rhythms could be associated with or even responsible for the multiple T_b_ rhythms observed in our study, but it is unclear why these rhythms would only be found in some individuals. Some individuals displayed diurnal activity patterns whereas others displayed nocturnal activity patterns, while others still switched between patterns within a cycle. While this variation is perplexing, these results are similar to previous work on locomotor activity of mole-rats in the laboratory [55], [56], suggesting the factor(s) driving these patterns is likely intrinsic and affects all aspects of rhythmicity.

Damaraland mole-rats are eusocial mammals with a distinctive reproductive caste based on dominance and body size and a secondary work related division of labour [57]. Dominance is linear and related to gender and body mass where the dominant male is the heaviest male in the colony and the dominant female is one of the heaviest individuals in the colony [57], [58]. The larger non-reproductive mole-rats comprising both sexes undertake little work and are referred to as infrequent workers (they spend <3% of time performing burrow maintenance) while the smaller non-reproductive individuals constitute a frequent worker group (they spend up to 15% of time performing maintenance) [57], [58]. Still, there is a strong positive relationship between body mass and energy expenditure [59], so it is interesting that body mass has a relatively small effect on variation in T_b_s in this species. Unfortunately, our dataset is not conducive to an evaluation of the role of social standing on T_b_, but there are numerous other physiological differences between infrequent and frequent workers [59], so it would not be surprising to find a relationship between caste and T_b_ variation.

Our study is the first to investigate T_b_ of a free-ranging southern African subterranean rodent species that has been continuously monitored for a considerable period of time and highlights the substantial individual variation in the T_b_ of free-ranging Damaraland mole-rats. Further, our study is one of relatively few to measure seasonal T_b_ patterns in small (i.e., less than 1 kg), highly homeothermic endotherms in the field [12], [13], [60], despite the fact that the majority of mammals and birds are homeothermic. Importantly, our results, and those of other studies on homeotherms [12], [13], [60], strongly support the prediction that the seasonal patterns of T_b_ in homeotherms should mirror those of heterotherms, but in a more muted fashion [1]. This evidence supports other studies that have shown homeothermic species display larger fluctuations in T_b_ when the cost of thermoregulation increases (e.g., [61]). Many studies have focused on rhythms of T_b_ in small homeothermic species (e.g., [51], [62]), and our results add to that body of literature while confirming that no universal T_b_ rhythms are likely to exist in mole-rats [55], [56]. Considerable future research is needed on the T_b_ patterns of homeothermic species in the wild, especially in the subtropics and tropical regions, where research is lacking.

## Supporting Information

Table S1 Example body temperature data for male and female Damaraland mole-rats (*Fukomys damarensis*) recorded during summer and winter in the Kalahari Desert, South Africa.
(XLSX)
